# Minimizing decompression and warming during deep seawater collection increases abundance and activity of autochthonous bacteria and archaea

**DOI:** 10.1093/ismejo/wrag064

**Published:** 2026-07-02

**Authors:** Alvaro M Plominsky, Logan M Peoples, Matthew Norenberg, Salvador Ramirez-Flandes, Sheila Podell, Kelli K Mullane, David Casagrande, Christopher Roman, Robert Pockalny, David C Smith, Caroline Belser, Julie Poulain, Eric E Allen, Ronnie N Glud, Osvaldo Ulloa, Nicholas Barber, Steven D’Hondt, Douglas H Bartlett

**Affiliations:** Marine Biology Research Division, Scripps Institution of Oceanography, University of California San Diego, La Jolla, CA 92037, United States; Marine Biology Research Division, Scripps Institution of Oceanography, University of California San Diego, La Jolla, CA 92037, United States; Institute of Geophysics and Planetary Physics, Scripps Institution of Oceanography, University of California San Diego, La Jolla, CA 92037, United States; Instituto Milenio de Oceanografía, Universidad de Concepción, Casilla 1313, Concepción 4070386, Chile; Center for Marine Biotechnology and Biomedicine, Scripps Institution of Oceanography, University of California, San Diego, La Jolla, CA 92037, United States; Marine Biology Research Division, Scripps Institution of Oceanography, University of California San Diego, La Jolla, CA 92037, United States; Graduate School of Oceanography, University of Rhode Island, Narragansett, RI 02882, United States; Graduate School of Oceanography, University of Rhode Island, Narragansett, RI 02882, United States; Graduate School of Oceanography, University of Rhode Island, Narragansett, RI 02882, United States; Graduate School of Oceanography, University of Rhode Island, Narragansett, RI 02882, United States; Génomique Métabolique, Genoscope, Institut François Jacob, CEA, CNRS, Univ Evry, Université Paris-Saclay, 91057 Evry Cedex, Evry, France; Génomique Métabolique, Genoscope, Institut François Jacob, CEA, CNRS, Univ Evry, Université Paris-Saclay, 91057 Evry Cedex, Evry, France; Marine Biology Research Division, Scripps Institution of Oceanography, University of California San Diego, La Jolla, CA 92037, United States; Center for Marine Biotechnology and Biomedicine, Scripps Institution of Oceanography, University of California, San Diego, La Jolla, CA 92037, United States; Nordcee and HADAL, Department of Biology, University of Southern Denmark, Odense M, 5230 M, Denmark; Danish Institute for Advanced Study (DIAS), University of Southern Denmark, Odense M DK-5230, Denmark; Tokyo University of Marine Science and Technology, Deptartment of Ocean Sciences, 4-5-7 Konan, Minato-ku, Tokyo 108-8477, Japan; Instituto Milenio de Oceanografía, Universidad de Concepción, Casilla 1313, Concepción 4070386, Chile; Departamento de Oceanografía, Universidad de Concepción, Casilla 160–C, Concepción 4070386, Chile; Department of Biology, San Diego State University, San Diego, CA 92182, United States; Graduate School of Oceanography, University of Rhode Island, Narragansett, RI 02882, United States; Marine Biology Research Division, Scripps Institution of Oceanography, University of California San Diego, La Jolla, CA 92037, United States

**Keywords:** deep sea, microbial oceanography, pressure, piezo-sensitive, piezophile, microbial activity, cell lysis, metagenomics, metagenome-assembled-genomes

## Abstract

The deep ocean hosts autochthonous pressure-adapted microorganisms that are unique to this environment, as well as allochthonous pressure-sensitive members transported from shallow depths by vertical advection and particle-sinking. However, conventional sampling instruments decompress and warm deep-sea samples during retrieval, potentially altering microbial properties when studied *ex situ*. Here, we assess this potential sampling bias by comparing seawater microbial communities collected with or without measures aimed at minimizing pressure and temperature effects. When compared to samples collected under pressurized conditions, conventional sampling (using Niskin bottles) was found to affect prokaryotic cells retrieved by reducing their total numbers, diminishing protein synthesis activity (>10%), and also causing overall shifts in the community composition. The most significant compositional change was a >20% decrease in metagenomic archaeal representation (TACK-group/*Thaumarchaeota/Nitrososphaerota*). Deep-sea bacterial groups had mixed responses to preserving pressure during retrieval, with some groups exhibiting higher representation when samples were maintained pressurized (e.g. members of the family *Pelagibacteraceae*, unclassified *Thiotricales, Thioglobaceae*, and *Chitinophagaceae*), whereas others increased their representation when decompressed (e.g. *Burkholderiaceae, Comamonadaceae*, and *Oxalobacteraceae*). This study reveals the existence of bias introduced by the complete decompression of samples retrieved with traditional instrumentation, as well as a decrease in overall bacterial activity when samples are completely decompressed during retrieval. Additionally, incubations lasting for >24 h were shown to transform the original prokaryotic community composition. Precautions addressing these effects are necessary to enhance the reliability of *ex situ* measurements and improve our understanding of deep-sea microbial ecology and biogeochemistry.

## Introduction

The majority of the deep sea presents low temperatures and elevated hydrostatic pressures. These environments are inhabited by autochthonous microbiota that thrive under these conditions [1, 2] and are the main drivers of deep-ocean biogeochemical cycles [3, 4]. Within the water column, samples are generally collected using Niskin bottles, either attached to a tethered rosette or deployed with autonomous vehicles [5, 6]. When using this approach, the collected microorganisms are fully decompressed and warmed to varying degrees during their ascent and recovery. These pressure and temperature (P–T) shifts have been shown to irreversibly impair [2, 7] and lyse [8, 9] autochthonous deep-sea piezophilic (pressure loving) microorganisms.

To complicate matters further, allochthonous pressure-sensitive (piezosensitive) microorganisms may sink to depths [10, 11] where the P–T conditions of the deep sea suppress their metabolism but do not necessarily prevent their reactivation upon decompression [11–13]. Various simulation-based studies mimicking particle descent from surface waters to hadal depths have shown that microorganisms associated with these particles begin to show reduced enzymatic activity as early as 8 MPa (corresponding to 800 m below sea level, mbsl) [14], followed by progressive changes in cell size [15] and a decline in microbial respiration and degradation processes between 10 and 40 MPa (corresponding to 1000–4000 mbsl), with complete suppression of respiration and significant shifts in community composition occurring at pressures of 60 MPa and above (typical of the hadal zone between 6000 and ~11 000 mbsl) [16, 17].

Although it is possible to obtain cultures of some strictly psychrophilic (low-temperature loving) and obligately piezophilic bacteria with partially warmed and fully decompressed samples [18–20], the extent to which such collection methods distort the original prokaryotic community remains unknown. Even temporary P–T alterations likely distort assessments of microbial abundance, activity, and community composition, with ramifications for assessments of their role in biogeochemical cycling [2, 11–13, 21–27]. As a result, two general strategies have been developed to address this issue: (i) *in situ* approaches that fix samples at depth prior to recovery, sometimes after an incubation period [12, 14, 21, 23–25]; and (ii) *ex situ* approaches that minimize P–T perturbations during recovery, as well as perhaps during some downstream processing steps [22, 26, 27]. The use of these devices has demonstrated the importance of reducing P–T changes from those present *in situ*. Still, the overall effects of P–T shifts during retrieval on deep-sea microbiota remain incompletely characterized. Here, deep-sea microbial communities collected with traditional sampling devices (i.e. Niskin bottles) were compared to communities collected with a thermally jacketed pressure-retaining sampler (PRS) [27] from the San Clemente Basin (2000 mbsl and 10 m above the seafloor), the Puerto Rico Trench (2500–8270 mbsl and 5 m above the seafloor), and the Atacama Trench (5400–7700 mbsl). We aimed to assess the effects of P–T shifts during sample retrieval on prokaryotic cell numbers, microbial community composition, protein synthesis (i.e. cellular activity), and the metabolic potential of the most affected prokaryotic groups. The results illustrate the biases introduced when conducting deep-sea microbiological investigations using traditional seawater sampling methodologies.

## Materials and methods

### Sample collection

**Figure 1:**
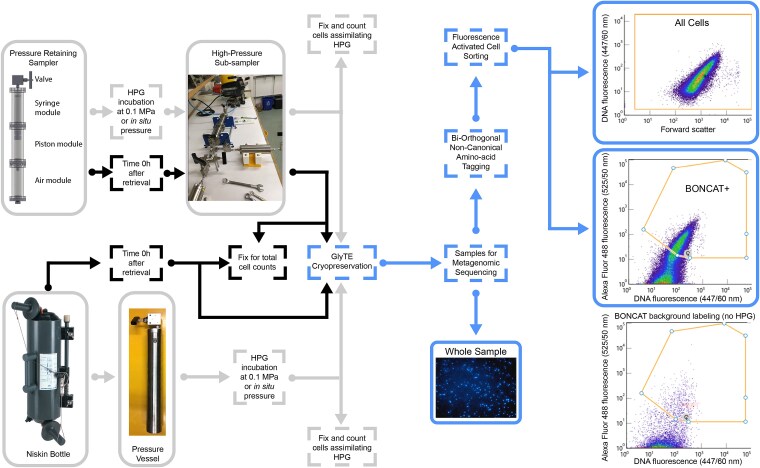
Deep-sea microbial sample processing. Detailed sample processing steps for HPG assimilating cells and total cell counts are denoted in grey and black arrows/squares, respectively. Processing steps and samples used for metagenomic sequencing are denoted in light-blue arrows and squares. “Whole sample” correspond to triplicate 50 ml aliquots, each filtered onto a 0.22 μm filter directly from the cryopreserved material, and then released with 200 μl of 0.025% (v/v) Tween 20 in PBS to be processed through MDA and DNA sequencing; “all cells” correspond to samples of cryopreserved material that were stained with a DNA dye, collected on a 0.22 μm filter, processed through the BONCAT labelling assay, and then sorted by flow cytometry, aiming to select events that presented the DNA dye; “BONCAT*+*” cell samples originated from the same material as ‘all cells’, but their sorting aimed for events that presented both the DNA dye and the fluorescent dye Alexa-Fluor 488 (attached to the HPG incorporated by the active cells). The selection of BONCAT*+* cells was established for each sample based on the level of BONCAT background labelling to the microbial community when not exposed to HPG, or when fixed before being exposed to HPG (i.e. reflecting the adhesion of the fluorescent label to cells that did not incorporate HPG).

Samples for microbial cell counts, metabolic activity, and metagenomic sequencing were collected at 2000 mbsl (10 m above the seafloor) in the San Clemente Basin during R/V *Gordon Sproul* 2018 cruise SP1821 (Supplementary Table S1 and Supplementary Fig. S1), at various depths in the Atacama Trench as part of the R/V *Sonne* 2018 cruise SO261 (Supplementary Table S2 and Supplementary Fig. S2), and various depths in the Puerto Rico Trench (5 m above the seafloor) during the R/V *Endeavor* 2019 cruise EN622 (Supplementary Table S3 and Supplementary Fig. S3). Samples were collected using Niskin bottles and PRSs capable of collecting and maintaining deep seawater samples at *in situ* pressure and thermally insulated [27]. The PRSs have a 316 stainless steel needle valve with an orifice size of 0.52 cm (High Pressure Equipment Co.) and is comprised of three modules (Fig. 1): a syringe module (housing a sterile polyetheretherketone syringe for collecting the sample of interest), a piston module (filled with sterile MilliQ water), and an air module [27]. The valve and first two modules are assembled while submerged in sterile ultrapure (MilliQ) water to avoid the presence of any compressible air space within these two modules. The valve is opened at the sampling depth by a remotely operated stepper motor actuator triggered simultaneously with the closing of the paired Niskin bottles. The deep (high-pressure) seawater enters the syringe cavity due to the pressure difference between the sampling location and the 0.1 MPa retained inside the PRS, by displacing the syringe piston and the MilliQ water within the syringe module. The displaced water then flows through the end-cap into the piston module and pushes a titanium piston, which in turn pushes the water within the piston module. Finally, the water in the piston module is displaced through the ruby nozzle (i.e. a synthetic ruby orifice inserted into a stainless steel housing that channels high-velocity fluids in one direction), located in the flange between the piston and air modules of the PRS (Fig. 1), which maintains a consistent and slow flow rate (the entire sampling process takes less than 1 min) and enters the air module. The water/air mixture in the air module is therefore sealed from the remainder of the pressurized PRS.

Simultaneous deployments of paired sets of PRSs and Niskin bottles in the *San Clemente Basin* were performed by sampling with an untethered “Leggo” lander system [27] carrying the PRS and both 30-l and 3.5-l Niskin bottles. The PRS was placed inside a casing filled with 30 l of ice-cold sterile MilliQ water prior to deployment to thermally insulate the PRS during its ascent. Seawater samples were collected ~10 m above the sediment–water interface, within the benthic boundary layer. In the Atacama Trench, sampling was performed using a Hadal–Rosette tethered to the ship frame of the R/V ‘Sonne’ [28] carrying the PRS and 20-l Niskin bottles. When sampling at the Puerto Rico Trench, seawater was collected using a Deep-sea Autonomous Profiler [29] carrying the PRS (inside a casing pre-filled with 30 l of ice-cold sterile MilliQ water) and 12-l Niskin bottles. Seawater samples were recovered ~5 m above the sediment–water interface within the benthic boundary layer.

All sampling systems were additionally equipped with conductivity-temperature-depth profilers. Lander instruments collected seawater samples while being static at their corresponding locations on the seafloor, and both the Niskin bottles and the PRSs were simultaneously triggered 2 h after reaching the seafloor to avoid capturing resuspended sediment material disturbed upon landing. Unless stated otherwise, the internal pressure of all PRS samples in this study was reinstated from the slightly diminished pressure upon retrieval to their *in situ* values immediately upon recovery.

### Subsampling at high pressure for its cryopreservation or fixation, and determination of microbial cell counts

To minimize the warming of the deep-sea samples, the PRSs were removed from the Hadal–Rosette or their insulated casing on the lander, and then placed on ice, taken to a 4°C room, and/or covered with chilled gel packs to keep them cold during sample processing. Similarly, samples collected using Niskin bottles were transferred to acid-washed, sterile brown High-Density Polyethylene (HDPE) bottles (Nalgene) to protect samples from the light and were kept on ice during sample processing. To avoid major shifts in hydrostatic pressure of the deep-sea samples, the PRSs were subsampled using a pressurized 5-ml syringe subsampling system inside a steel casing (Fig. 1 and Supplementary Fig. S4) [27] that maintained hydrostatic pressure for both the PRS and the subsamples while samples were either fixed for cell counts or embedded in glycerol TE for their posterior cryopreservation. During the Atacama Trench cruise, a sterile concentrated formaldehyde solution (3.5% w/w final concentration, Sigma-Aldrich) or a concentrated solution (resulting in a 5 μM final concentration) of the alkyne-containing methionine analog L-homopropargylglycine (HPG; Vector Laboratories) was loaded in the PRSs before their deployment. Upon retrieval, all samples were immediately fixed using a sterile, concentrated formaldehyde solution (final concentration 3.5% w/w; Sigma-Aldrich) while preserving *in situ* hydrostatic pressure using the steel subsampling system (see Fig. 1; Supplementary Fig. S4). Niskin bottle samples were transferred from the HDPE bottles to cryovials and then fixed with the same sterile, concentrated formaldehyde solution (final concentration 3.5% w/w; Sigma-Aldrich). Triplicate 2 ml aliquots of each fixed sample were flash-frozen and stored at −80°C until analysis. Prior to cytometry, samples were thawed on ice and stained with Hoechst 33342 (16.2 μM final concentration; Invitrogen) to specifically label DNA. To estimate absolute cell concentrations, we recorded all events in a defined volume of sample (Tables S1–S3). Cell counts were performed by flow cytometry on a ZE5 Cell Analyser (Bio-Rad), which detected Hoechst fluorescence (excitation: 355 nm; emission: 447/60 nm) to distinguish DNA-containing cells. Cytometry files were processed with FlowJo v10.5.3 (Becton, Dickinson and Company; 2019), applying a gate to exclude debris and noise: the threshold was defined just above 95% of events in negative-control samples (unfixed MilliQ water processed identically as the experimental samples). This approach ensured rigorous discrimination of true cells from background artefacts.

### Bi-orthogonal non-canonical amino acid tagging assessment of the proportion of active microbial members

In this procedure, the alkyne-containing methionine analog HPG is used to label newly synthesized proteins within cells after its bonding to a fluorescent reporter molecule (e.g. 488 Alexa Fluor azide dye) and thus allows the identification of cells that were active during the incubation period. For samples obtained during the San Clemente Basin cruise, a concentrated HPG solution (5 μM final concentration) was added inside the PRS containing the samples during re-pressurization upon retrieval. For Niskin bottle samples, a 100 ml aliquot was placed into sterile KAPAK bags (Komplete Packaging) and amended with a 5 μM final concentration of HPG from the Click-iT kit (Invitrogen) and heat sealed. Samples were then incubated for 48 h at 4°C either at atmospheric or *in situ* pressure in stainless steel pressure vessels [19, 30]. To measure non-specific fluorescent labelling of cells, triplicate negative controls for microbial activity consisting of 20 ml aliquots taken from the PRSs, were transferred into sterile 100 ml KAPAK bags (Komplete Packaging), and fixed for 15 min at room temperature with a sterile concentrated formaldehyde solution (3.5% w/w final concentration, Sigma-Aldrich) before adding a concentrated solution of HPG (Click Chemistry Tools) for 5 μM final concentration, and incubated for 48 h at 4°C and atmospheric (0.1 MPa) pressure.

One PRS sample at 7000 mbsl in the Atacama Trench and all Puerto Rico Trench PRSs were deployed with a known volume of sterile concentrated HPG solution (5 μM final concentration once the deep-sea sample was collected). To determine the background labelling of cells, negative bi-orthogonal non-canonical amino acid tagging (BONCAT) controls were assessed using samples not exposed to HPG that were collected with Niskin bottles simultaneously and from the same water mass as the PRSs. These controls were never exposed to HPG before being fixed for 15 min at room temperature with a sterile concentrated formaldehyde solution (3.5% w/w final concentration, Sigma-Aldrich) and later processed through the same BONCAT-labelling procedure as the PRS samples.

All experimental samples for cell counts were fixed for 15 min (using the high-pressure sub-sampler for the PRS samples [27]) with a sterile concentrated formaldehyde solution (3.5% w/w final concentration, Sigma-Aldrich) and then stored at −80°C until further processed (Fig. 1). Samples were thawed on ice, stained with Hoechst 33342 trihydrochloride trihydrate (16.2 μM final concentration, Invitrogen), and filtered onto 0.22 μm pore-size filters (25 mm, GTTP, Millipore) before subjecting them to the copper-catalysed click-reaction to bond the Alexa Fluor 488 azide through BONCAT [31, 32]. San Clemente Basin samples were labelled using the Click-iT kit (Invitrogen) following the manufacturer’s instructions. Samples from the Atacama and Puerto Rico trenches were labelled as described in the BONCAT coupled with fluorescence activated cell-sorting procedures (BONCAT-FACS; see Supplementary Materials and Methods).

Total and (active) HPG-containing cells were quantified by flow cytometry using a ZE5 Cell Analyser (BioRad). Hoechst 33342 (excited with a 355 nm laser and its emission detected on a 447/60 nm bandpass filter) and Alexa Fluor 488 fluorescence (excited with a 488-nm laser and its emission detected on a 525/50-nm bandpass filter) events were recorded in parallel. Triplicate BONCAT assays from each location and pressure condition were performed to determine the percent of active cells in each sample. All the events with Hoechst 33342 signal were considered to be the *“*total cells” whereas only cells presenting both Alexa Fluor 488 and Hoechst 33342 signal were classified as “active cells” in each sample. Flow cytometry data files, used to calculate the percentage of active cells in each sample, were analysed using FlowJoTM software v10.5.3 (Becton, Dickinson and Company). Lasers were always aligned and calibrated before running samples with 3 μm ultra-rainbow fluorescent beads (Spherotech Inc.).

### Metagenomic read processing and taxonomic assignment of unassembled reads

The quality of all raw metagenomic sequences was assessed with FastQC v11.9 [33] and processed accordingly using Trimmomatic v1.2.14 [34]. The unassembled metagenomic reads (>150 bp) were taxonomically classified by determining their similarity to single-copy genes and J, A, K, and L categories from the Cluster of Orthologous Genes v2020 database [35] with DIAMOND [36] using the “--ultra-sensitive” setting. The metagenomic reads associated with information storage and transfer COGs (JAKL) were used for taxonomic assignments to increase the resolution of this assessment and because these functional categories are known to have lower rates of horizontal gene transfer than other categories [37, 38]. This is a pragmatic simplification based on the current understanding of the “complexity hypothesis” [37], which postulates that the number of protein–protein interactions of a gene product determines its transferability [38]. The reads associated with contaminant taxa identified in our negative BONCAT-FACS control samples (Supplementary Materials and Methods) were removed from downstream analysis, and the resulting count and taxonomy data were further processed using PhyloSeq [39]. Community similarity/dissimilarity metrics were calculated using group average’s hierarchical cluster analysis based on Bray–Curtis dissimilarity matrix with the proportional abundances of the metagenomic reads related to single-copy genes and JAKL COG categories with the Vegan package v2.6-5 [40] in R v3.6.1.

Community taxonomic profiles were also assessed using all unassembled reads. These were taxonomically assigned with Kaiju v1.7.3 [41] using the NCBI RefSEQ complete genomes v2020-05-25 without Eukaryota as a reference database (Supplementary Fig. S6). All the read processing and assemblies were performed in the KBase environment [42] within the narrative https://doi.org/10.25982/90888.1452/2327015. This Kbase narrative and all the corresponding data and parameters are publicly available.

More details on methods and sample processing can be found in Supplementary Materials and Methods regarding: Biomass Fractionated Community DNA extractions and sequencing, BONCAT labelling procedures for cell-sorting of active prokaryotes, BONCAT-FACS and multiple displacement amplification of active prokaryotes, identifying contaminants and generation of metagenome assembled genomes (MAGs), construction of site-specific pan-genomes and determining their metabolic potential, statistical analyses assessing the effects of decompression during retrieval on prokaryotic cell numbers, and data visualization.

## Results

### Pressure and temperature shifts decrease deep-sea microbial cell counts

**Figure 2:**
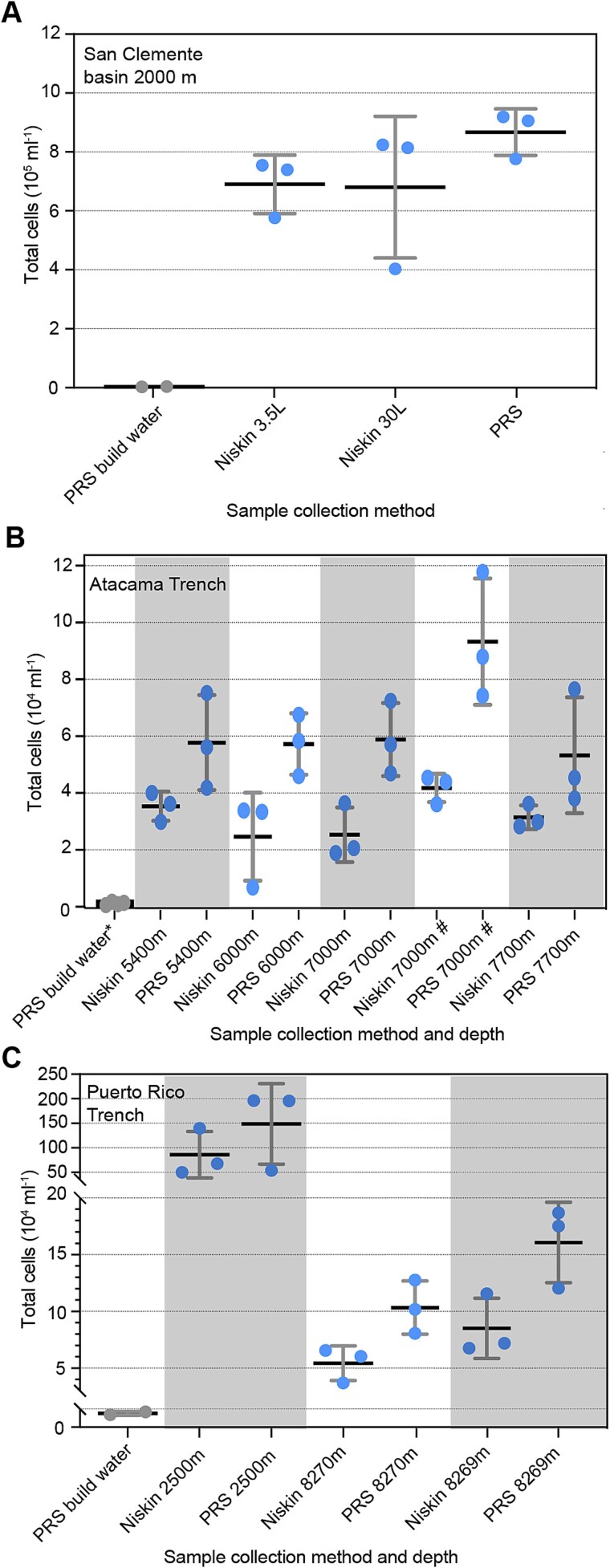
Cell counts utilizing different retrieval instrumentation. Flow cytometry total cell counts for samples retrieved simultaneously with Niskin bottles or PRSs from: (A) the San Clemente Basin 2000 mbsl; (B) the Atacama Trench (all PRS samples were fixed *in situ*, except for cast “7000 m #” which was fixed once retrieved); and (C) the Puerto Rico Trench. Each value corresponds to three independent flow cytometry experiments for each deployment and their arithmetic mean (± SD). Also, the microbial cells detected in the MilliQ water after the manipulation of the PRS during its assembly (“PRS build water”) are shown (*n* = 2 or 3). Shapiro–Wilk test for Gaussian distribution and statistical comparison of cell counts for each sample are available in Supplementary Table S12.

To assess how traditional methods affect the total number of prokaryotic cells, bathypelagic samples from the San Clemente Basin were sampled (collected at 2.62°C from 2000 mbsl) (Supplementary Table S1) using an autonomous lander equipped simultaneously with thermally insulated PRSs [27] (each with 150 ml of internal sampling volume), and Niskin bottles of 3.5 l and 30 l. The PRSs lost <30% (6 MPa) of the *in situ* pressure (20 MPa) and limited the temperature increase to 7.2°C in surface waters of 19.8°C. Unless stated otherwise, the internal pressures of all the PRS samples in this study were reinstated to their *in situ* values upon retrieval and processed maintaining their *in situ* pressure using a high-pressure subsampler (Fig. 1 and Supplementary Fig. S4) [27]. The samples collected with 30-l Niskin bottles were retrieved at the same temperature as the PRS but completely decompressed to atmospheric pressure (0.1 MPa). In contrast, samples collected with 3.5-l Niskin bottles were both decompressed to atmospheric pressure and warmed to 18°C during recovery. All Niskin bottle samples had similar total cell counts (arithmetic mean of 6.90 × 10^5^ cells ml^−1^, SD ±0.99 × 10^5^ for 3.5-l Niskin; and 6.80 × 10^5^ cells ml^−1^, SD ±2.40 × 10^5^ for 30-l Niskin), which although not statistically significant when comparing to their paired PRS samples these Niskin samples were on average 20% lower than those from samples retrieved simultaneously with the PRS (8.67 × 10^5^ cells ml^−1^, SD ±0.8 × 10^5^; Kruskal–Wallis test, *P*-value = .1679; Fig. 2A, and Supplementary Table S1). A similar sampling approach was performed to compare the influence of decompression during retrieval among various abyssal and hadal samples in the Atacama Trench (Supplementary Table S2). In this case, samples were collected simultaneously using 12-l Niskin bottles and PRSs (which lost 31%–50% of their *in situ* pressure during retrieval). All Atacama Trench Niskin bottle samples were fixed upon retrieval, while PRS instruments were preloaded with a concentrated solution to obtain a final concentration of 3.5% (w/w) formaldehyde. One of the Atacama Trench PRS deployments (from 7000 mbsl) was instead preloaded with an (innocuous) synthetic amino acid to label the active members of the community (see section on deep-sea microbial activity below), and a subsample of it was fixed upon retrieval for cell counts. Regardless of *in situ* fixation, the average total cell counts of all the Atacama Trench samples collected with the PRSs were over 38% higher when compared to the samples retrieved with Niskin bottles (Fig. 2B). Deep-sea samples from the Puerto Rico Trench (Supplementary Table S3) were also simultaneously collected using either 12-l Niskin bottles or the PRSs (which lost 30–38% of their *in situ* pressure) to further assess the influence of decompression during sample retrieval. The results of this cruise showed that all the samples collected with the PRSs possessed over 42% higher average total cell counts compared to their paired Niskin bottle retrievals (Fig. 2C). This effect was consistent across all deep-sea locations, and quasipoisson generalized linear modelling (Supplementary Materials and Methods) indicated that the average PRS cell counts were significantly greater than the average Niskin bottle cell counts across all depths (*P* < .001; incidence rate ratio = 1.55, 95% confidence interval [1.26, 1.90]). Consistently, a generalized linear mixed-effect model incorporating deployment as a random effect (and accounting for overdispersion) produced the same qualitative outcome (Supplementary Materials and Methods), with PRS cell counts remaining significantly higher than those from Niskin bottles. However, in pairwise comparisons, the variation among replicates was large in all cases, which resulted in the higher average total PRS cell counts compared to their paired Niskin bottle retrievals being statistically significant only among some hadal samples (*P* < .05, paired two-tailed *t*-test; Supplementary Table S12). This variation among replicates was consistently observed for both the PRS and Niskin bottle samples and might reflect how uneven each one of them was affected by the freeze/thaw and manipulation procedures. The fact that higher prokaryotic cell numbers were observed regardless of whether or not *in situ* fixation occurred in the samples collected with the PRSs suggests that the lower numbers of cells in samples collected with Niskin bottles were due to decompression-dependent cell lysis during their ascent (Fig. 2).

### Decompression during retrieval affects the resulting deep-sea microbial community composition

The taxonomic composition of deep-sea microbiota was assessed using paired PRS/Niskin bottle sampling. Triplicate “whole samples” corresponding to all the cells in the cryopreserved material simultaneously retrieved using each instrument (Fig. 1) were processed through multiple-displacement amplification (MDA), and then each set of replicates was pooled for metagenomic sequencing (Supplementary Materials and Methods). Additionally, in the San Clemente Basin, a portion of the water simultaneously retrieved using Niskin bottles was size fractionated to generate traditional shotgun metagenomes for the biomass fractions of >3 μm, and from 0.2–3 μm (Supplementary Table S4). Similar size-fractionated metagenomes (Supplementary Materials and Methods) were generated for two 7000 mbsl Atacama Trench samples (Supplementary Table S4). These traditional size-fractionated shotgun metagenomes served as references to assess the impact of MDA [43] on the PRSs and Niskin bottle ‘whole samples’.

**Figure 3:**
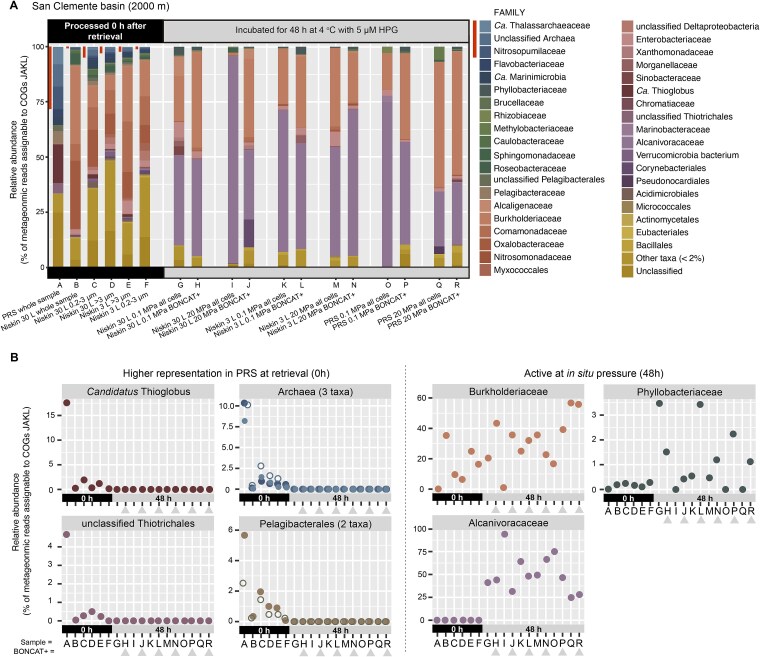
Family-level taxonomic profiles of deep-sea microbiota retrieved from the San Clemente Basin with different instrumentation. (A) Classification of all unassembled metagenomic reads associated with genes from COG JAKL categories (Supplementary Table S6) of samples retrieved from 2000 mbsl in the San Clemente Basin immediately upon collection and after an incubation at and 4°C and either *in situ* or atmospheric pressure. The additional vertical lines inserted to the left of the bars are meant to highlight archaeal groups in each plot and denote the relative abundances of archaea in each sample. For each metagenome, the bottom labels detail the sampling method (PRS or Niskin bottle), and either the biomass size fraction from which DNA was extracted (0.22 to 3 μm, or > 3 μm) or the cell selection strategy (“whole sample” = metagenomes of the complete samples before BONCAT-FACS; “all cells” = samples processed for BONCAT-FACS that were stained with DNA dye; “BONCAT+” = cells stained with both DNA and the azide dye, indicative of active protein synthesis). (B) Relative abundances of key microbial groups that present higher representation 0 h after retrieval or those shown to be active after being incubated for 48 h at *in situ* pressure are detailed. Only taxa representing >2% in at least one of the metagenomes were plotted (otherwise they were agglomerated as “other taxa”).

**Figure 4:**
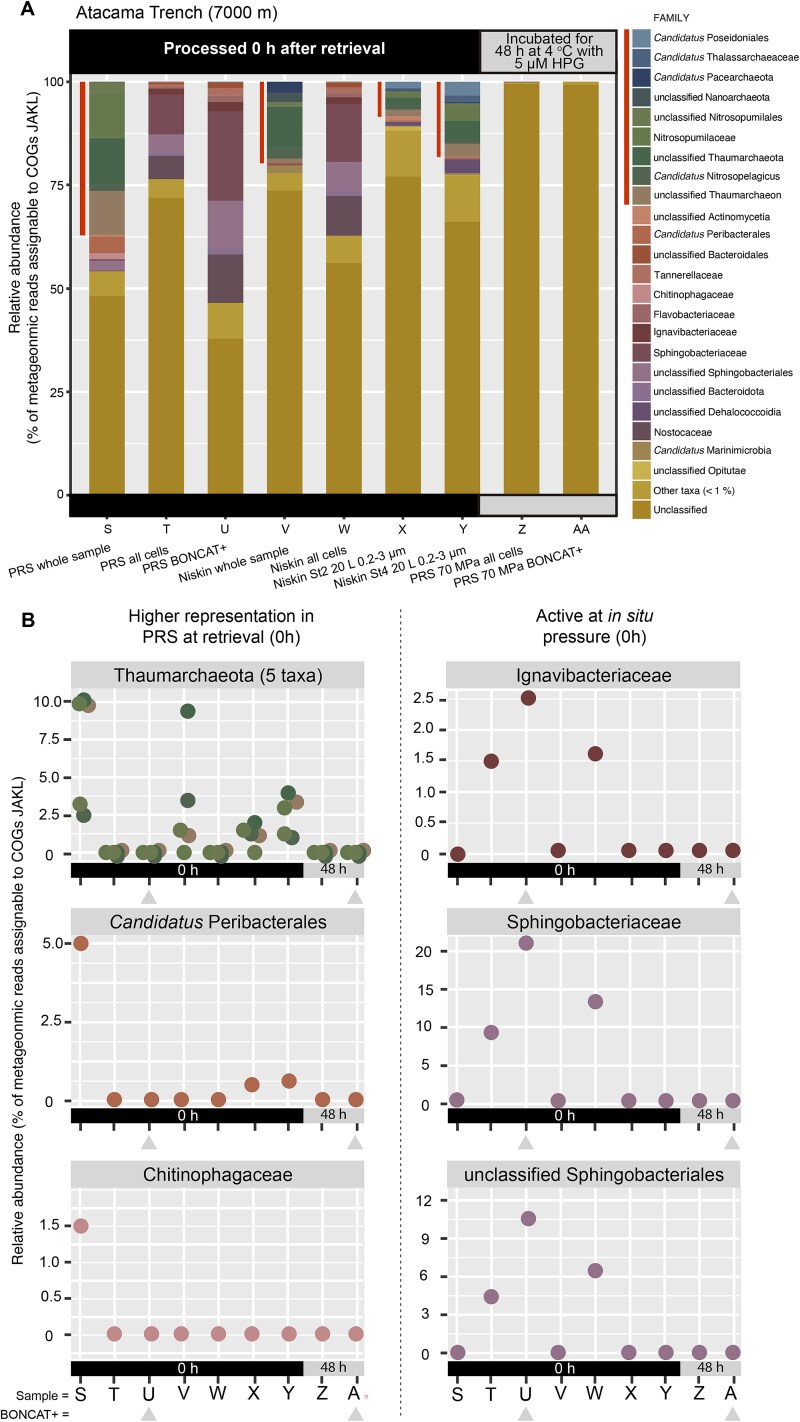
Family-level taxonomic profiles of deep-sea microbiota retrieved from the Atacama Trench with different instrumentation. (A) Classification of unassembled metagenomic reads associated with genes from COG JAKL categories (Supplementary Table S8) of samples retrieved from 7000 mbsl in the Atacama Trench immediately upon collection and after an incubation of 48 h at *in situ* pressure and 4°C. The red vertical lines inserted to the left of the bars are meant to highlight archaeal groups in each plot and denote the relative abundances of archaea in each sample. For each metagenome, the bottom labels detail the sampling method (PRS or Niskin bottle), and either the biomass size fraction from which DNA was extracted (0.22 to 3 μm) or the cell selection strategy (“whole sample” = metagenomes of the complete samples before BONCAT-FACS; “all cells” = samples processed for BONCAT-FACS that were stained with DNA dye; “‘BONCAT+” = cells stained with both DNA and the azide dye, indicative of active protein synthesis). (B) Relative abundances of key microbial groups that upon retrieval present higher representation in the PRS compared to the Niskin bottles and those that when processed through BONCAT were shown to be active after being incubated with HPG during their ascent. Only taxa representing >1% in at least one of the metagenomes were plotted (otherwise they were agglomerated as “other taxa”).

Because COGs from “information processing” and “single-copy” genes are known to have lower horizontal transfer rates than other metabolic categories [37, 38], the unassembled metagenomic reads associated with these categories were used to taxonomically profile the deep-sea communities. The San Clemente Basin 2000 mbsl samples retrieved with the Niskin bottles (i.e. both size-fractionated and ‘whole sample’) presented a similar taxonomic composition (Fig. 3A and Supplementary Fig. S5A and S6A). However, the PRSs ‘whole sample’ had twice the Bray–Curtis distance to all the samples simultaneously retrieved using Niskin bottles (“A” compared to “B,” “C,” “D,” “E,” and “F”; Supplementary Fig. S7A). One of the major differences in the taxonomic composition among these San Clemente Basin samples was a six-fold greater metagenomic representation of Archaea (Thaumarchaeota-Aigarchaeota-Crenarchaeota-Korarchaeota TACK-group/*Thaumarchaeota/Nitrososphaerota*) for samples retrieved with the PRSs compared to Niskin bottles (29% compared to <5%; Fig. 3A and B and Supplementary Fig. S5A). At the family level, the representation of *Thioglobaceae* (from 17% to <2%), *Pelagibacterales* (from 8% to <4%), and *Thiotricales* (from >4% to <0.5%) were reduced in the decompressed Niskin bottle compared to the PRS samples (Fig. 3A and B and Supplementary Fig. S5C). Instead of Archaea, representation of the bacterial phyla Proteobacteria/Pseudomonadota, Verrucomicrobia (PVC-group), and Terrabacteria was higher in decompressed Niskin bottles compared to PRS samples (Supplementary Fig. S5A). Specifically, the families that had higher metagenomic representations in the Niskin bottles compared to the PRS samples were *Burkholderiaceae* (from 0.08% to between 6% and 35%), *Comamonadaceae* (from undetectable to between 7% and 23%), and *Oxalobacteraceae* (from undetectable to between 8% and 30%) (Fig. 3A and Supplementary Fig. S5C). Similar results were observed for metagenomes from 7000 mbsl in the Atacama Trench (Fig. 4A). The PRSs ‘whole sample’ presented a higher metagenomic representation of the phylum *Thaumarchaeota/Nitrososphaerota* (38% compared to <15%), Candidatus Peregrinibacteria (5% compared to <2%), and the Bacteroidota/Bacteoidetes family *Chitinophagaceae* (1.5% compared to <0.005%) compared to the decompressed Niskin bottle ‘whole sample’ collected simultaneously and the two size-fractionated metagenomes from the Atacama Trench axis (Fig. 4B and Supplementary Fig. S5B and D). Instead, the Atacama Trench Niskin bottle metagenomes had a higher representation of DPANN-group and Euryarchaeota (>2.5% compared to <0.1%) and Proteobacteria/Pseudomonadota (>19% compared to <9%) than the PRS ‘whole sample’ (Fig. 4A and Supplementary Fig. S5B). Moreover, taxonomic distributions for all the decompressed Niskin bottle metagenomes clustered together with twice the Bray–Curtis distance from the community sampled using the PRS (“S” compared to “V,” “X,” and “Y”; Supplementary Fig. S7B).

Overall, maintaining the samples pressurized resulted in the retrieval of distinct microbial taxa compared to when samples were exposed to complete decompression (Supplementary Fig. S5E and F). Similarly, the same trends were observed when comparing the microbial composition of these San Clemente Basin and Atacama Trench metagenomes sampled with the PRS or Niskin bottle using the taxonomic assignment of their unassembled reads associated with any translated gene sequence in the NCBI RefSeq database (Supplementary Fig. S6).

### Complete decompression and warming decreases deep-sea microbial activity

**Figure 5:**
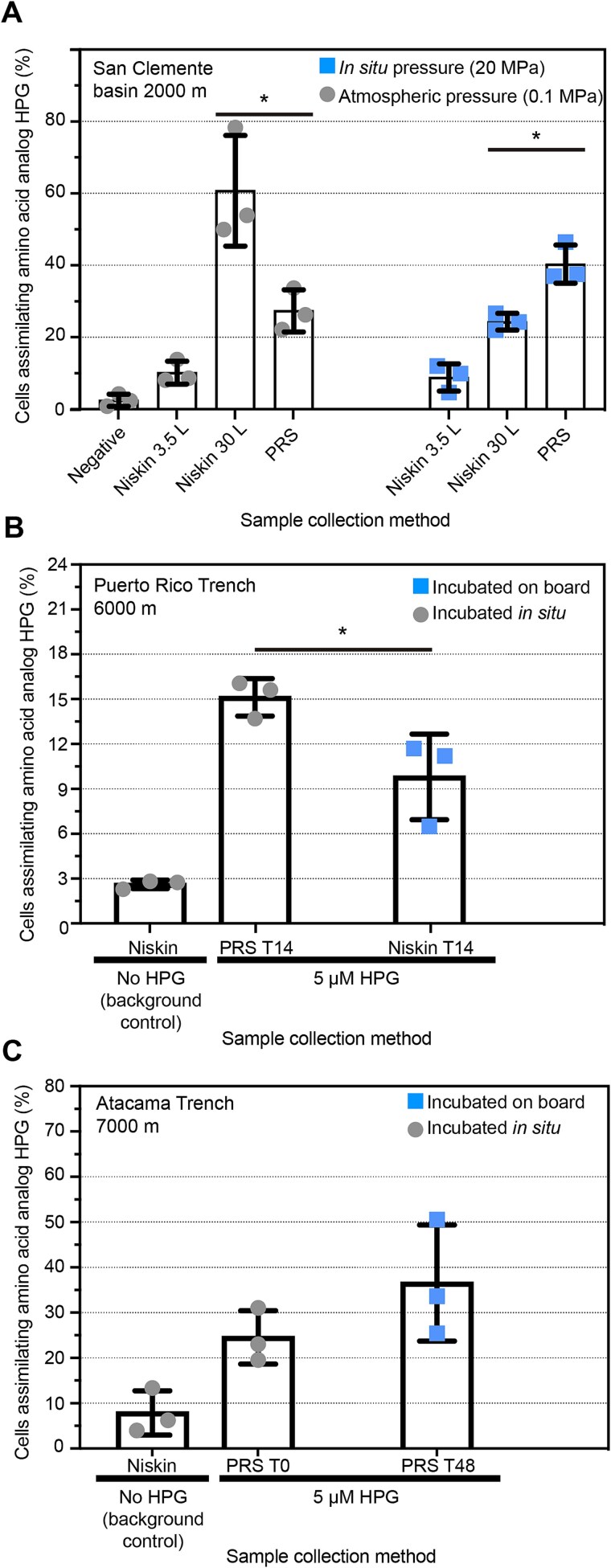
The influence of sample collection methods on deep-sea microbial activity. (A) Percent of active cells in microbiota from 2000 mbsl in the San Clemente Basin retrieved simultaneously with Niskin bottles or PRSs after 48 h incubation at 4°C under atmospheric (◯) or *in situ* (▪) pressure (*n* = 3 independent BONCAT experiments; Supplementary Fig. S4). Negative controls, reflect the background labelling of the Alexa 488 azide dye to fixed biological material. (B) Percentage of active cells in microbiota from 6000 mbsl in the Puerto Rico Trench retrieved simultaneously with Niskin bottles and PRSs 0 h after retrieval (◯) or after a 14 h onboard incubation at 4°C under *in situ* (▪) pressure (*n* = 3 independent BONCAT and flow cytometry experiments; Supplementary Fig. S6 and Supplementary Table S15). (C) Percent of active cells in microbiota from 7000 mbsl in the Atacama trench retrieved with the PRSs 0 h after retrieval (◯) or after a 48 h onboard incubation at 4°C under *in situ* (▪) pressure (*n* = 3 independent BONCAT and flow cytometry experiments; Supplementary Fig. S5). Statistically significant values (*P* < .05; detailed values for normality, ANOVA, and multiple comparisons tests are available in Supplementary Table S16) are denoted in each graph, indicating the samples compared (“*”).

BONCAT [31, 32] was used to determine how decompression during the collection of deep-sea samples affects the proportion of (active) community members synthesizing new proteins. However, when comparing the community composition of the Atacama Trench ‘whole sample’ and BONCAT-FACS ‘all cells’ (compare “S” with “T,” and “V” with “W” in Fig. 4), it was evident that this procedure greatly affected archaeal community members. As a result, the BONCAT-FACS results have only been presented for the bacterial members of the communities examined. In the San Clemente Basin, all samples were amended with the synthetic amino acid HPG immediately after retrieval and incubated at 4°C for 48 h either at atmospheric (0.1 MPa) or *in situ* (20 MPa) pressure (Fig. 1 and 5A, Supplementary Fig. S8). These experiments revealed that the highest overall fraction of active bacterial cells was present in the large Niskin bottle sample incubated at atmospheric pressure, which was greater than that of the same seawater sample repressurized to *in situ* pressure or that of the decompressed PRS sample also incubated at atmospheric pressure. The second greatest fraction of active bacterial cells was present in the PRS sample incubated at the *in situ* pressure, which was greater than that of the same seawater sample decompressed to atmospheric pressure or the repressurized Niskin bottle sample. Thus, sampling with the 30-l Niskin bottle (decompressed, but thermally insulated during retrieval) increased the representation of bacteria that were active when incubated at atmospheric pressure, whereas retrieval with the PRS significantly favoured bacteria capable of synthesizing new proteins at *in situ* pressure. These effects could not be reproduced by decompressing pressure-retained samples or repressurizing decompressed samples, respectively.

To determine the effects of decompression on the metabolic activity of hadal microbiota, a PRS preloaded with a concentrated solution of HPG was deployed at 6000 mbsl in the Puerto Rico Trench. This PRS opened at the sampling depth, closed after it had filled, and was then left to incubate “on-site” for 14 h at a potential temperature of 2°C before being retrieved (losing 40% of its *in situ* pressure during recovery). Triplicate samples were simultaneously retrieved with 12-l Niskin bottles. These were re-pressurized immediately upon retrieval and incubated with HPG for the same 14 h at 4°C on board (Fig. 1). The sample temperatures upon retrieval were 6.5°C and 12.1°C for the PRS and Niskin bottles, respectively. The PRS microbiota incubated on-site had a significantly higher proportion of metabolically active bacterial cells than those simultaneously sampled with Niskin bottles, re-pressurized, and incubated on board (*P* < .05, one-way ANOVA and Sidak’s multiple comparisons tests; Fig. 5B and Supplementary Table S15). Overall, these results together with the decompression-dependent loss of prokaryotic cells described above suggest that a significant proportion of deep-sea bacterial piezophiles and/or piezo-tolerant bacteria (i.e. that are metabolically active *in situ*) are lost when samples completely decompress and/or warm during retrieval.

### Long-term incubations reshape deep-sea microbiota samples

Although re-pressurization and incubation in sealed containers are traditionally utilized to enrich and isolate deep-sea prokaryotes [30, 44], this procedure increases metagenomic bacterial representation and reduces archaeal representation and overall diversity [7, 22, 24, 26] by selecting for members that tolerate the enclosure and/or its amendments (namely “Bottle Effect”) [45–49]. Additionally, the BONCAT cell labelling process, and cell selection by FACS performed at atmospheric pressure also biased the representation of the communities present in ‘all cells’ compared to ‘whole sample’ metagenomes (“S” compared to “T,” and “V” compared to “W”; Fig. 4A).

Here, parallel samples retrieved with PRSs or Niskin bottles were incubated for 48 h at 4°C with HPG (Fig. 1). Then, cells were labelled with BONCAT and differentiated using FACS for either all the microbial cells (“all cells”) or only those that incorporated HPG after 48 h (“BONCAT*+*”; Fig. 1 and Supplementary Fig. S9 and S10). Regardless of the pressure, the incubation procedure led *all cells* from both the 2000 mbsl San Clemente Basin and the 7000 mbsl Atacama Trench samples to shift their community composition, and in the case of the latter to become dominated by the families *Alcanivoracaceae* and *Burkholderiaceae* within the Proteobacteria/Pseudomonadota phyla. These groups also constituted the majority of the “*BONCAT+*” cells among the incubated samples (Fig. 4, Supplementary Fig. S5 and S6). The MAGs generated from the San Clemente Basin incubations were taxonomically assigned to the species *Alcanivorax xenomutans* (family Alcanivoracaceae), *Achromobacter insuavis*, and *Paraburkholderia fungorum* (both from the *Burkholderiaceae* family). The hydrocarbonoclastic *Alcanivorax* genus has been shown to bloom after exposure to alkanes and various plastics [50]. The San Clemente Basin *A. xenomutans* population posessed various transporters for peptides, amino acids, and urea in addition to a nitrite reductase gene (*nirK*, K00368; Supplementary Fig. S11; Supplementary Table S26). The two *Burkholderiaceae* populations that dominated the enclosed samples are ubiquitous across soil, freshwater, and marine environments [51, 52]. Although many members of the *Achromobacter* and *Paraburkholderia* genera are nonpathogenic they can outcompete host-associated microbiota to establish persistent biofilms [53, 54]. Both of these *Burkholderiaceae* populations from the San Clemente Basin present the metabolic potential for denitrification (Supplementary Fig. S11; Supplementary Table S26). The potential for anaerobic metabolisms fueled by the reduction of nitrate and the known capacities of these microorganisms to dominate environmental and host-associated environments could explain their increased representation among the San Clemente Basin “enclosed” samples.

To determine if short incubations were a valid alternative to accurately assess the active microbial members from deep-sea environments, a PRS preloaded with a concentrated solution of HPG was deployed to 7000 mbsl in the Atacama Trench and its active prokaryotes were labelled during their (4 h) ascent. The proportion of active bacteria that were labelled during the 4 h period (T0, processed 0 h after retrieval) was not statistically different from that observed after the same PRS was incubated on board for an additional 48 h at 4°C (*P* > .05, one-way ANOVA and Sidak’s multiple comparisons tests; Fig. 5C). The 7000 mbsl Atacama Trench T0 samples were subjected to BONCAT-FACS-MDA to generate metagenomes and identify if there were groups with a greater metagenomic representation among cells taking up HPG (“*BONCAT+*”) during the 4 h incubation at *in situ* pressure (Fig. 1). This assessment revealed that sequences assigned to (Bacteroidetes/Bacteroidota) members of the family *Ignavibacteriaceae* and *Sphingobacteriales* had a higher proportion of sequences in the T0 PRS “*BONCAT+*” compared to both the PRS *all cells* and Niskin bottle *all cells* metagenomes (“U” compared to “T” and “W” in Fig. 4B and Supplementary Table S8). These findings are consistent with a previous study showing that the PRS enabled the retrieval of single-cell genomes of Bacteroidetes/Bacteroidota representatives from the Challenger Deep, at 10970 mbsl in the Mariana Trench, that were specifically enriched in hadal zones and had high 16S rRNA gene identity to other sequences retrieved from deep-sea locations, and in particular trenches [27]. Therefore, short incubations (of at least 4 h) allowed assessment of the active microbial members in a setting that did not bias the community (Fig. 4A and Supplementary Fig. S5C).

### What is the metabolic potential of prokaryotes that become underrepresented (or even undetected) when using traditional instrumentation?

**Figure 6:**
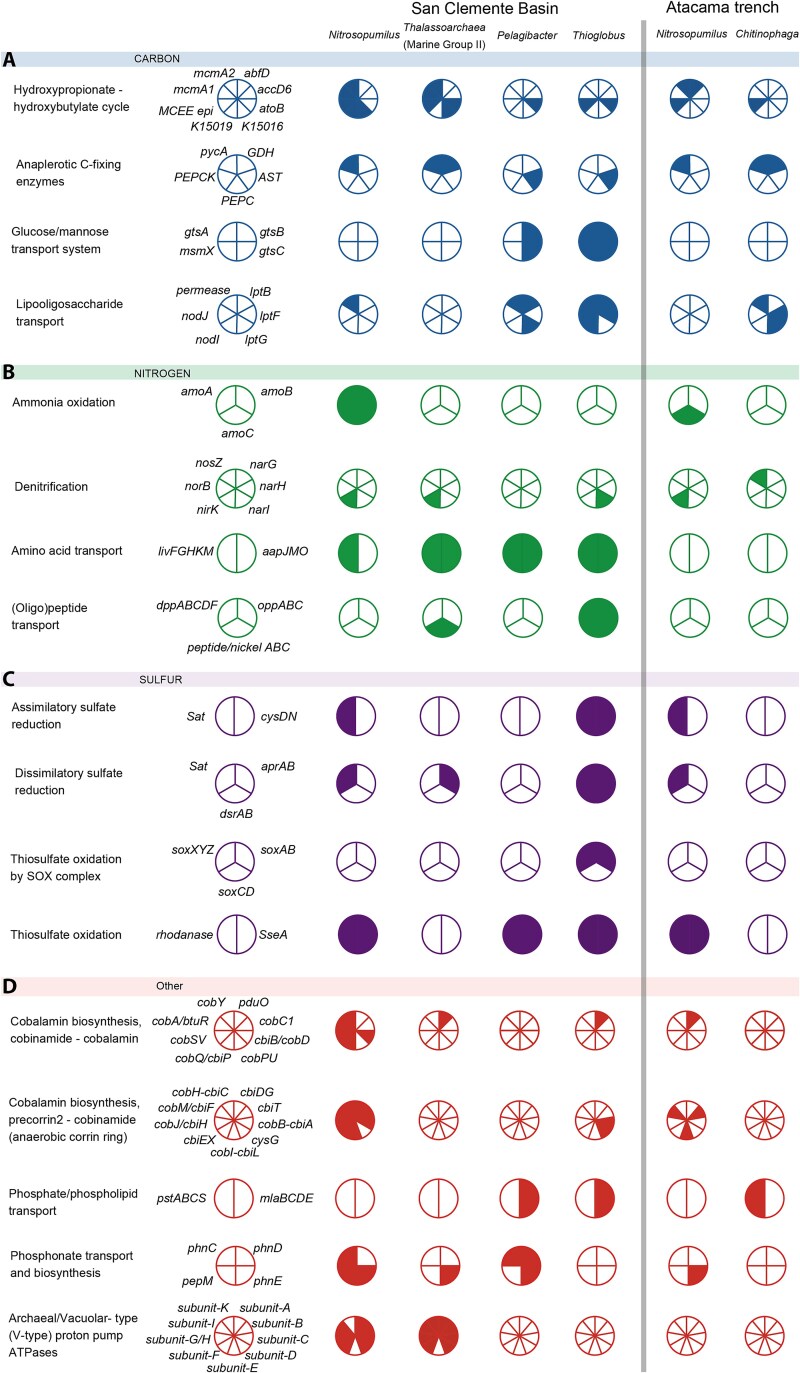
Metabolic potential of deep-sea site-specific pangenomes of groups that become underrepresented when decompressed during retrieval. The (genus-level) pangenomes are listed across the top with pathways associated with (A) carbon, (B) nitrogen, (C) sulfur, and (D) “other” metabolisms provided below as rows. The presence of genes assigned to a specific function was compiled and classified using their distilled and refined annotation of metabolism [68] as guides. The filled regions of each circle denote the presence of a specific gene/subunit of the corresponding pathway or transporter (as specified in the key pie chart located on the left side, Supplementary Table S25). Since all the samples from T0 were sampled simultaneously and came from the same water parcel, we combined both the T0 metagenomes from the PRS and the Niskin bottles to have a more sequencing coverage for this (genus-level) pangenome effort.

The metabolic contributions of microbial lineages affected by complete decompression during retrieval may be underrepresented when using traditional sampling methodologies. An initial attempt to assess the metabolic potential of the most dominant populations affected by decompression through traditional metagenome contig binning only resulted in mostly low- to medium-quality MAGs (Supplementary Table S18) that encompassed <50% of all the contigs assigned to each group of interest (Supplementary Tables S19–S24). For this reason, the metabolic potential of these microbial groups was assessed by an alternative method (Supplementary Materials and Methods), first taxonomically classifying all individual metagenomic contigs, and then grouping them into site-specific genus-level pan-genomes (Supplementary Tables S19–S24). The San Clemente Basin pan-genomes corresponded to members of the genus *Nitrosopumilus, Thalassoarchaea* (Marine Group II), *Pelagibacter*, and *Thioglobus*. From the Atacama Trench, pan-genomes from the genus *Nitrosopumilus* and *Chitinophaga* were further analysed. Information on the carbon, nitrogen, sulfur, vitamin, and phosphorus metabolisms present in genus-level pan-genomes of these groups is provided in Fig. 6 and Supplementary Table S25. These results denote the metabolic potential of at least a subset of the members from these taxa that might be underrepresented when subjected to complete decompression during the sampling of these deep-sea environments.

Various genes coding for subunits of V-type ATPases (Fig. 6D), a suggested phylogenetic marker for piezophilic species [55], were present in both the San Clemente Basin *Nitrosopumilus* and *Thalassoarchaea* pan-genomes. Although no genes for V-type ATPases were detected in the hadal Atacama Trench *Nitrosopumilus* (Fig. 6D), this might be due to the smaller total number of genes recovered in this pan-genome (676 genes; Supplementary Table S23) compared to the San Clemente Basin *Nitrosopumilus* and *Thalassoarchaea* pan-genomes (3554 and 7573 genes, respectively; Supplementary Tables S19, S20).

The presence of *nosZ* genes in the San Clemente Basin *Chitinophaga* pan-genome (Fig. 6B) supports the potential of this genus to perform nitrous oxide reduction, thereby expanding to marine environments the involvement of this Bacteroidetes/Bacteroidota genus in nitrogen loss [56, 57].

## Discussion

Seawater sampling is arguably the most basic process in all of oceanography. Niskin and PRS sampling have in common the collection of a contiguous mass of seawater, but differ in input orifice size, internal volume, and *in situ* pressure maintenance during recovery. Among these, avoiding complete decompression appears to be the most critical [2, 4, 11, 12, 20–24], at least until bottle effects develop. The effects of decompression are also likely enhanced by the warming of microbial communities adapted to living at ~3°C during retrieval [7], as observed for the 3.5-l Niskin bottle sample from 2000 mbsl in the San Clemente Basin, which warmed to 18°C during its recovery. In this case, microbial activity was not significantly different from negative controls fixed before the addition of HPG, when incubated at either pressure (*P*-value >.05, one-way ANOVA and Sidak’s multiple comparisons tests; Fig. 5A).

One indication of the effects of complete decompression is the higher cell numbers obtained with the PRS instruments regardless of whether or not the PRS samples are fixed *in situ*, with the extent of these differences tending to increase with depth (Fig. 2). The increasing difference in cell counts between PRS and Niskin samples with depth suggests that deep-sea microbes may be more vulnerable to decompression. We propose that greater pressure sensitivity at greater depths contributes to the amplified disparities in cell recovery. Future studies combining pressure-retaining sampling with taxon-specific viability or membrane integrity assays could help determine whether these depth-related trends reflect shifts in community composition with varying susceptibility to decompression.

Because the Niskin samples were never fixed, their increased prokaryotic activity induced by decompression [12] could lead to increased viral productivity and its resulting impacts on cell numbers. However, our experiments cannot distinguish if the difference in cell counts among these instruments is because the PRS reduces the extent of phage- and/or decompression-induced cell lysis that occurs in Niskin bottle samples. A prior study revealed a similar trend in microscopy cell count differences with Niskin/PRS recovery using the same Atacama Trench samples examined in our study. It also provided cell and virus flow cytometry abundances in syringe samples fixed *in situ* compared to onboard [28]. It concluded that any bias in abundances induced by sample recovery from hadal depth was smaller than sample-to-sample variations [28]. Although we cannot compare our results directly to the different sampling technique involving syringes, a significant difference between the previous study and our current assessment is that when the average differences between Niskin/PRS samples were analysed using flow cytometry rather than epifluorescence microscopy, they revealed a statistically significant trend.

The cell counts from 2000 mbsl in the San Clemente Basin and 2500 mbsl in the Puerto Rico Trench were overall 2–10 fold higher than values reported for the same depths in the Mariana and Japan Trenches [46, 58, 59]. These higher values could be due to these sampling sites being closer to land, located only 40 nmi and 30 nmi offshore for the San Clemente Basin and Puerto Rico Trench, respectively. Another possibility could be the influence of cells from the benthic boundary layer [60] or those that were resuspended from the sediment at the moment the landers reached the seafloor. In this regard, it may be noteworthy that although no turbidity/backscatter traces are available, there was no evidence of sediment particles in any of the San Clemente Basin and Puerto Rico Trench flow cytograms. Consistently, all our abyssal and hadal cell counts were within the same ranges as described in a parallel effort assessing the same sampling of Atacama Trench locations of this study [28].

Deep-sea Archaea appear to be particularly sensitive to decompression and downstream processing steps. Consistent with our results, a previous study assessing bathypelagic ammonia-oxidizing *Thaumarchaeota/Nitrososphaerota* populations from the Mediterranean Sea [20] showed that there were compositional changes among these groups when sampling involved 2 h of decompression using traditional Niskin bottles compared to *in situ* filtration and 1 h of decompression prior to on-board fixation. As described for pelagic [7] and benthic [61, 62] deep-sea microbial communities, another condition that could bias cell abundance and activity is the steepness of the temperature gradients in the water column. For example, cells in higher-latitude environments experience less temperature change throughout the water column.

BONCAT is a powerful technique for the identification and selection of active cells [31, 32]. However, we note two limitations in its application. First, shifts in microbiota composition shown here and in previous studies for deep-sea microbial incubations spanning >24 h [7, 22, 24, 26] emphasize the need for shorter activity labelling periods to reduce bottle effects (Fig. 3 and Fig. 4). One way to accomplish this is to label cells during their recovery. We found that effective BONCAT labelling of active cells from 7000 mbsl PRS samples could be performed during a 4-hour ascent, with no additional shipboard incubations required (Fig. 5C).

The second limitation is that our BONCAT-FACS activity profiling proved to be more useful for select bacterial community members while reducing the representation of Archaea present in the deep-sea samples examined here. This, despite the fact that BONCAT has been successfully applied to a variety of archaeal groups [31, 32]. Some deep-sea bacterial groups also shifted their representation when subjected to the BONCAT-FACS process when comparing Atacama Trench samples that were processed 0 h after retrieval (compare “S” with “T,” and “V” with “W” in Fig. 4). Because this (Fig. 3 and Fig. 4) and previous studies [7, 46] have been able to extract archaeal DNA from size fractionated deep-sea Niskin samples from cells collected on filters, one explanation for the decrease of these taxa among the Atacama Trench PRS and Niskin ‘all cells’ compared to their ‘whole sample’ metagenomes could be cell lysis due to exposure to certain chemicals (e.g. copper ions and Tween 20 detergent) during the BONCAT labelling procedure.

Another important conclusion from this study is that repressurizing seawater samples collected with decompression does not compensate for the effects of decompression. Complete decompression during retrieval with Niskin bottles significantly reduces the proportion of bacterial members that are capable of synthesizing new proteins at their *in situ* P–T, whereas greatly enhancing their activity at atmospheric pressure (Fig. 5A and B). Although our study here focused on seawater samples from depths beyond 2000 mbsl, a previous study [63] indicated that this is also true for microbial communities from shallower waters at 1100 mbsl (i.e. decompression during sample collection using traditional instrumentation can alter measurements of microbial activity). These results and those of a recent study [12], that compared bathypelagic *in situ* prokaryotic heterotrophic production to production in decompressed samples, illustrate the complexity of pressure-driven responses across distinct deep-ocean niches and the effects of decompression on allochthonous and autochthonous microorganisms inhabiting deep-sea environments. The results presented here provide cell-count, bacterial activity, and metagenomic assessments that highlight how the interplay between decompression-sensitive and pressure-tolerant microbial populations may shape activity patterns under different pressure retrieval regimes. These findings provide context for interpreting *in situ* measurements compared to decompressed measurements [12], suggesting that the differential recovery of pressure-sensitive taxa may partly explain the apparent overestimation of metabolic activity under atmospheric conditions. Furthermore, the genomic capabilities of microbial taxa detected exclusively in the PRS samples (i.e. such as sulfate and nitrate reduction) suggest these communities may be sediment- and/or particle-associated rather than strictly planktonic. Their presence, particularly in bathyal waters where anomalously high cell abundances were observed, likely reflects recent inputs of rapidly sinking organic matter derived from seasonal surface productivity or the “funneling” of sidewall particulates within trenches. Taken together, these observations also support a scenario in which microbial populations from shallower waters were transported to depth attached to particulate material. In Niskin bottle samples, the depressurization, temperature increase, and delay before fixation may have stimulated metabolic activity in these allochthonous populations, including elevated viral replication and subsequent cell lysis. Thus, the results of this study are consistent with decompression and increasing temperatures that could derive in two possible effects: (i) the lysis of high-pressure and low-temperature-adapted autochthonous deep-sea prokaryotes, and/or (ii) prophage induction and cell lysis in shallow-water allochthonous prokaryotes. These mechanisms could account for the pronounced cell loss observed in decompressed samples and align with the interpretations proposed in previous studies [12]. Regardless of the relative importance of these two influences, avoiding decompression during deep-sea sample retrieval is important for accurate assessments of their microbial community structure and function.

The pressure effects noted above have ramifications for downstream analyses of the microbial biogeochemical and metabolic potential. The genome-informed properties of the microbial groups negatively affected by the P–T shifts with traditional instrumentation from the San Clemente Basin (sampled at 2000 mbsl and 10 m above the seafloor) and Atacama Trench included pathways involved in carbon fixation, ammonium oxidation, nitrogen loss, oxidation of sulfur compounds, and the usage/synthesis of phosphonates (Fig. 6).

In summary, evidence has been presented from several ocean basins that the abundance, structure and activity of microbial seawater communities observed in the “piezosphere” are sensitive to the extent of decompression experienced during recovery, a result with major implications for assessments of microbiota at depth. While the extent of depressurization may be the dominant factor, cell recovery is likely to be a function of changes in pressure, changes in temperature, and time to cell fixation. Additional experiments involving additional locations, depths, and water masses will be required to develop a more comprehensive view of these impacts. These should ideally involve comparisons of identical seawater samples distinguished only by their extent of decompression. Future efforts to identify microbial groups from geographically distinct regions affected by sample decompression during recovery and their biogeochemical impacts would benefit from employing complementary methodologies to those used in this study. For example, traditional fluorescence *in situ* hybridization labelling [64] and quantitative PCR [65] targeting groups shown here to be affected by decompression or untargeted approaches like 16S rRNA gene amplicon sequencing of samples retrieved with and without decompression are also an interesting alternative. However, the latter would require a careful selection of primers since some have limited success in amplifying certain deep-sea groups of interest (i.e. Archaea) [66] compared to others [7, 67]. Alternatively, by using fixatives that preserve the transcriptional profile of deep-sea microbiomes, future studies could unveil the *in situ* metabolism of these communities. This could include a more systematic application of *in situ* samplers/measurements [12, 14, 21, 23–25], and/or the use of *ex situ* P–T retention approaches [22, 26, 27]. In either application, steps to reduce bottle artefact effects will also be critical for obtaining taxonomically and metabolically accurate assessments of deep-sea microbial communities.

## Supplementary Material

Supplementary_material_wrag064

## Data Availability

Raw and processed (meta)genomic data are publicly available through NCBI short read archive through BioProject PRJNA1069756 (acc. no. SAMN39621693-SAMN39621728) and ENA BioProject PRJEB57853 (acc. no. ERS3170393 and ERS3170398), and the Kbase narratives http://dx.doi.org/10.25982/90888.1452/2327015 and http://dx.doi.org/10.25982/138628.97/2327013.
