# Supplementary material for: Minimizing decompression and warming during deep seawater collection increases abundance and activity of autochthonous bacteria and archaea
Source: ISME J. 2026 Jul 2;20(1):wrag064. doi: 10.1093/ismejo/wrag064 (PMC13332712; doi:10.1093/ismejo/wrag064)
Supplement: Supplementary_material_wrag064 [file supplementary_material_wrag064.zip › Plominsky_et_al_Suppl_Mat_Met_2025-12-18.docx]

**Supplementary Materials and Methods**

Biomass Fractionated Community DNA extractions and sequencing

The San Clemente Basin 2,000 mbsl samples (Supplementary Table S4) collected with 3.5- and 30-L Niskin Bottles were placed on ice and sequentially filtered >2 L of each sample through 3-μm (47 mm, polycarbonate, Millipore) and 0.22-μm pore size filters (47 mm, GTTP, Millipore) using a peristaltic pump (ChemTec Model 1082, SciLog BioProcessing Systems) at a starting flow rate of ~80 mL/minute. Samples were maintained between 4 ^o^C and 7 ^o^C during the filtration procedure by maintaining seawater samples in ice and lines covered with chilled gel packs. Filters were flash frozen in dry ice and then stored at -80 ^o^C until further processed. DNA was extracted by cutting the filters with sterile surgical blades and then using the Quick-DNA Miniprep Kit (Zymo) “*solid tissue*” procedure. The quality of the community DNA was revised using a LabChip GX Touch nucleic acid analyser (Perkin Elmer). Sequencing libraries were built using ThruPLEX DNA-Seq HV chemistry (Takara), size selected with a PippinHT (Sage Science).

The Atacama Trench 7,000-mbsl samples from station 2 and station 4 (Supplementary Table 4) were collected using 12-L Niskin Bottles attached to a custom-made Hadal Rosette [28]. Approximately 10 L of seawater were pre-filtered through a 20-mm mesh and then the microbial community was serially concentrated onto a 3-μm filter (polycarbonate, Millipore) and onto a 0.22-μm filter (polyethersulfone, Millipore), respectively. The filters were stored in liquid nitrogen until the arrival to the laboratory and then kept at -80 °C. The filters containing the microbial samples were delivered to the French National Sequencing Center - Genoscope (Évry, France). There, DNA extraction (method ID: Acinas_prok_DNA_extr), and sequencing library preparation (method ID: MetaG [4.1]) were carried out as previously described [72]. Briefly, the filters containing the microbial samples were cut into pieces and soaked in 3 mL of lysis buffer (40 mM EDTA, 50 mM Tris-HCl, 0.75 M sucrose). Lysozyme (1 mg mL^−1^ final concentration) was added and samples were incubated at 37 °C for 45 min while slightly shaken. Then, sodium dodecyl sulfate (1% w/v final concentration) and proteinase K (0.2 mg mL^−1^ final concentration) were added and samples were incubated at 55 °C for 60 min while slightly shaken. The lysate was collected and an equal volume of Phenol:Chloroform:Isoamyl Alcohol (25:24:1 v/v) was added, and then carefully mixed and centrifuged 10 min at 3,000 *g*. The aqueous phase was recovered and the procedure was repeated once. An equal volume of Chloroform:Isoamyl Alcohol (24:1 v/v) was added to the recovered aqueous phase in order to remove residual phenol. The mixture was centrifuged and the aqueous phase was recovered for further purification. The aqueous phase was then concentrated and purified by centrifugation with a Centricon concentrator (Amicon Ultra-4 Centrifugal Filter Unit with Ultracel-100 membrane, Millipore). Once the aqueous phase was concentrated, the DNA was washed three times by passing 2 mL of sterile MilliQ water. Libraries were prepared using the ‘on beads’ protocol of the NEBNext DNA Sample Prep Master Mix kit (New England Biolabs). Ligation was performed with adapted concentrations of Nextflex DNA barcodes (Revvity Inc.) and cleaned up by two rounds of AMPure XP beads purifications.

All the samples from the Atacama Trench and San Clemente Basin were sequenced using Sp500 chemistry (PE250, Illumina) on a NovaSeq 6000 instrument (Illumina).

BONCAT labelling procedures for cell-sorting of active prokaryotes

Cryopreserved samples were thawed at 4 ^o^C and then stained with Hoechst 33342 trihydrochloride trihydrate (16.2 µM final concentration, Thermo Fisher Scientific). Then, a dye solution was made by mixing 5 μL copper sulphate (CuSO4, 100 μM final concentration; Sigma-Aldrich), 10 μL tris-hydroxypropyltriazolylmethylamine (THPTA, 500 μM final concentration; Click Chemistry Tools), and 3.3 μL (Alexa Fluor 488 picolyl-azide, 5 μM final concentration; Click Chemistry Tools). This dye solution was incubated at room temperature for 3 min in the dark before mixing it with the reaction buffer solution consisting of 50 μL sodium ascorbate (Sigma-Aldrich) freshly prepared in PBS (5 mM final concentration), 50 μL of aminoguanidine HCl (Sigma-Aldrich) freshly prepared in PBS (5 mM final concentration), and 880 μL of PBS. Microbial cells were concentrated onto 0.22 µm pore-size filters (13 or 25 mm diameter, GTTP, Millipore) and washed three times by gently passing 2 mL of fresh PBS through the filter. The filters were then placed on a glass slide and 80 μL of the click reaction solution was added on top before covering the filter with a coverslip. The slides were incubated at room temperature for 3 min in the dark before removing each filter and triple-washing them again with 2 mL of PBS each time. The filters were then transferred, with the cells facing inwards, to 1.5 mL centrifuge tubes with 1 mL of 0.025 % (v/v) Tween 20 in PBS. Tubes were vortexed (Vortex-Genie 2, Scientific Industries) at half speed for 5 min to detach the cells and then stored at 4 ^o^C (for no more than 12 h) till used for FACS.

BONCAT-FACS, and multiple displacement amplification of active prokaryotes

Sample collection and processing methods for assessing the proportion of active microbial members were the same as described above, except for the following modifications. At zero hours after retrieval or after their corresponding incubation period, samples and negative controls were amended with a sterile concentrated solution of glycerol in TE buffer (10 % v/v final concentration) [73] for 15 min at 4 ^o^C (using the high-pressure sub-sampler for the PRS samples) and then stored at -80 °C until further processed (Fig. 1). Samples were thawed on ice and then three types of processing strategies were applied to the San Clemente and Atacama Trench samples (Fig. 1):

“***Whole samples***”: triplicate 50 mL aliquots filtered onto a 0.22-μm filter directly from the cryopreserved material, and then released from the filter with 200 μL of 0.025 % (v/v) Tween 20 in PBS to be processed through MDA and DNA sequencing.

“***All cells***”: triplicate samples of cryopreserved material were stained with a DNA dye, and then collected on a 0.22-μm filter and processed through the BONCAT labelling assay. These samples were then sorted by flow cytometry, aiming to select any events that presented the DNA dye (>3,000 cells were sorted for each sample). After cell-sorting, the resulting samples were processed through MDA and subjected to DNA sequencing.

“***BONCAT+***” cells: these samples originated from the same material as ‘*all cells*’ samples, but their cell sorting aimed for events that presented both the DNA dye along with the fluorescent dye Alexa-Fluor 488 attached to the HPG incorporated by the active cells. Thus, these samples are composed solely of prokaryotes that incorporated the synthetic amino acid into proteins synthesised during the incubation period.

Prokaryotes that incorporated HPG were labelled by BONCAT as described previously [74] (see above).

Then, HPG-containing cells were separated by FACS using a MoFlo Astrios EQ flow cytometer (Beckman Coulter). Cell selection was triggered by gating for events that presented both Hoechst 33342 (excited with a 355 nm laser and emission detected on a 448/59 nm bandpass filter) and Alexa Fluor 488 fluorescence (excited with a 488 nm laser and emission detected on a 513/26 nm bandpass filter). In addition to cells presenting both Hoechst 33342 and Alexa Fluor 488 fluorescence (i.e. ‘*BONCAT+*’, microbial cells that incorporated HPG), total microbial cells (*i.e.*, ‘*all cells*’, all events with Hoechst 33342 fluorescence) were also sorted (Fig. 1). To minimise the introduction of microbial contaminants during this procedure, the flow cytometer fluid lines and sheath fluid reservoir were washed with 10 % (v/v) bleach before performing the FACS, and the sterile PBS used as sheath fluid was passed through a 0.22-μm Sterivex filter unit (Millipore) installed along the fluid lines before entering the flow cytometer. A control sample consisting of the sterile MilliQ water used to build the PRSs was subjected to the BONCAT-FACS procedure to identify microbial contaminants derived from sample processing. Lasers were always aligned using 3 μm ultra-rainbow fluorescent beads (Spherotech, Inc.) before running samples.

The REPLI-g Advanced DNA Single Cell kit (Qiagen) was used to perform all Multiple Displacement Amplification (MDA) reactions following previously described procedures for microbial samples^45^. Triplicate BONCAT-FACS experiments were performed for each sampling location and their sorting strategies (*i.e*., ‘*whole cells*’, ‘*all cells*’, ‘*BONCAT+*’, and negative control sample types; Fig. 1). Input volumes containing the same number of "sorted cells" from each set of triplicate PRS and Niskin sample types were processed in parallel through MDA, using the same REPLI-g kit to process all the experimental samples and negative controls, and then pooled at equimolar concentrations for DNA sequencing. Sequencing libraries were built with the same methods and reagents as the biomass-fractionated DNA extraction (Supplementary Materials and Methods), using ThruPLEX DNA-Seq HV chemistry (Takara), size selected with a PippinHT (Sage Science), and sequenced using Sp500 chemistry (PE250) on a NovaSeq 6000 instrument (Illumina).

Identifying contaminants and generation of Metagenome Assembled Genomes (MAGs)

To increase the sequencing depth for the construction of MAGs, the metagenomic reads from the San Clemente Basin 2,000 mbsl or Atacama Trench 7,000 mbsl samples processed 0 h after their simultaneous retrieval (*i.e*. obtained from the same seawater mass during the same deployment but collected either with Niskin bottles or PRSs) were co-assembled. The corresponding quality-filtered metagenomic reads were combined into a single file with Merge Reads Libraries v1.0.1 [42]. Control samples from the processing steps of the BONCAT-FACS-MDA procedures were also combined as a distinct set. Each combined read library was then assembled with MetaSPAdes v3.13.0 [75] using a K-mer size of 33 and keeping only contigs >2Kbp. Initial contig bins were obtained with MaxBin2 v2.2.4 [76] (using both the default 107 bacterial marker gene set option, and the 40 bacterial and archaeal marker gene set), MetaBAT2 v1.7 [77], and CONCOCT v1.1 [70]. These preliminary bins were refined with DASTool v1.1.2 [69]. MAGs were assessed for quality using CheckM v1.0.18 [71], and taxonomically assigned with the Genome Taxonomy DataBase-ToolKit (GTDB-tk v1.7.0 and GTDB R06-RS202 release)[78, 79]. This procedure identified contaminants in our negative BONCAT-FACS-MDA control samples from the genera *Pseudomonas* and *Alcaligenes* (Gammaproteobacteria), as well as *Mesorhizobium* (Alphaproteobacteria) and *Flavobacterium* (Bacteroidota)(Supplementary Table 17). Additionally, DECONTAM [80] was used to further identify contaminants within these metagenomic datasets using the “frequency” method with a default probability threshold (TRUE if p < 0.1) to discriminate foreign taxa observed in the control MilliQ water sampled from each cruise and processed throughout the same BONCAT-FACS-MDA procedures as the DNA sourced from seawater samples.

All the assemblies and GTDB-tk analysis were performed in the KBase environment [42] within the narrative https://doi.org/10.25982/90888.1452/2327015. Contig bins assigned to the contaminating lineages were removed from the San Clemente Basin 2,000 mbsl and Atacama Trench 7,000 mbsl samples T0 metagenomes using Fractionate Contigs v1.2.0 (https://github.com/kbaseapps/kb_AssemblyUtilities) implemented within the KBase environment [42] in the narrative <https://doi.org/10.25982/138628.97/2327013>.

MAGs of >25% completeness were created to assess the metabolic potential of microorganisms dominating San Clemente Basin 48h incubations using the same procedure detailed above (Supplementary Fig. 11). The metabolic potential of the DAStool MAGs was complemented with that obtained when using CONCOCT v1.1 [70] because it generated the outputs with least contamination and highest completeness of each individual binning program according to their CheckM v1.0.18 [71] assessments (narrative https://narrative.kbase.us/narrative/213608).

Construction of site-specific pan-genomes and determining their metabolic potential

The San Clemente Basin 2,000 mbsl and Atacama Trench 7,000 mbsl T0 DAStool MAGs were of only low- to medium-quality (Supplementary Table S18), failing to capture the overall metabolic potential of the deep-sea microbial groups of interest. Therefore,

non-contaminant contigs from all the T0 metagenomes were taxonomically classified using DarkHorse version 2.0_rev09 ([http://darkhorse.ucsd.edu](http://darkhorse.ucsd.edu/)) [81] based on a Diamond version 2.0.15 [36] blastp search of all of their predicted proteins against GenBank nr (database accessed January 2019). An overall lineage was assigned to each contig (Supplementary Tables S19-24) based on a consensus of top database matches for its predicted proteins, as previously described [82]. The contigs of microbial lineages of interest (*i.e*., the microbial taxa whose representation decreased significantly when pressure and temperature were not maintained during sample collection; Fig 3 and Fig. 4) were identified and separated to build site-specific pan-genomes (Supplementary Tables S19-24). These sequences were re-uploaded to the KBase environment and processed through the Annotate and Distill Assemblies with DRAM v0.1.2 [68] implemented within the KBase environment [42] in the narrative https://doi.org/10.25982/138628.97/2327013.

Data visualisation

Graphs were visualised using ggplot2 v3.3.6 (ref. 78) in R v3.6.1 (ref. 79), or Prism v8.4.3 (GraphPad Software, LLC.).

Statistical analyses assessing the effects of decompression during retrieval on prokaryotic cell numbers

Two hypotheses were tested, first that PRS sample cell counts were greater than Niskin bottle samples, and second that this difference increased at greater sampling depth. Generalised linear models were used, specifying poisson error family and log link function for count data, and quasi poisson to account for overdispersion. Cells per mL were rounded to the nearest integer, and predictors were sampler type (PRS or Niskin), site (Atacama Trench, Puerto Rico Trench, or San Clemente Basin), sample depth, and the sampler x depth interaction. Analysis was carried out in R, and predictors were tested using type III likelihood ratio tests with Anova in the car package [84]. The interaction was nonsignificant, so we removed it and tested predictors in the additive model using type II tests. When removing the “depth interaction”, and testing “sampler type” across “sampling location” in an additive model for all the abyssal and hadal depths together, it verified that the average PRS cell counts were significantly greater than Niskin bottle cell counts across depths (*i.e*., the minimum difference was 20% more counts in the PRS samples, but on average PRS cell counts were 55% greater than Niskin bottle cell counts considering all depths sampled in this study). To evaluate whether our conclusions were robust to potential non-independence among samples collected during the same deployment, we fitted a generalized linear mixed-effects model (GLMM) with a Poisson error distribution. Deployment was included as a random intercept, and an observation-level random effect was added to account for overdispersion. Fixed effects initially included sampler type, depth, site, and the sampler × depth interaction. The interaction term was not significant (χ² = 1.88, df = 1, P = 0.170) and was therefore removed, after which an additive model was evaluated. As in the non-mixed generalized linear model, sampler type remained highly significant (χ² = 41.00, df = 1, P < 0.001), with PRSs yielding higher cell counts than Niskin bottles across all samples. Depth (χ² = 29.66, df = 1, P < 0.001) and site (χ² = 41.15, df = 2, P < 0.001) were also significant predictors. Overall, the GLMM results were qualitatively identical to those obtained with the non-mixed model.

SUPPLEMENTARY REFERENCES

69. Sieber CMK, Probst AJ, Sharrar A et al. Recovery of genomes from metagenomes via a dereplication, aggregation and scoring strategy. Nat Microbiol 2018;3:836–43. https://doi.org/10.1038/s41564-018-0171-1

70. Alneberg J, Bjarnason BS, de Bruijn I et al. Binning metagenomic contigs by coverage and composition. Nat Methods 2014;11:1144–6. https://doi.org/10.1038/nmeth.3103

71. Parks DH, Imelfort M, Skennerton CT et al. CheckM: assessing the quality of microbial genomes recovered from isolates, single cells, and metagenomes. Genome Res 2015;25:1043–55. https://doi.org/10.1101/gr.186072.114

72. Alberti A., et al. Viral to metazoan marine plankton nucleotide sequences from the Tara Oceans expedition. *Sci. Data* 2017;4.1:170093. https://doi.org/10.1038/sdata.2017.93

73. Rinke C. et al. Obtaining genomes from uncultivated environmental microorganisms using FACS-based single-cell genomics. *Nat. Protoc*. 2014;9:1038–48. https://doi.org/10.1038/nprot.2014.067

74. Couradeau E. et al. Probing the active fraction of soil microbiomes using BONCAT-FACS. *Nat. Commun*. 2019;10:2770. https://doi.org/10.1038/s41467-019-10542-0

75. Nurk S, Meleshko D, Korobeynikov A, Pevzner PA. metaSPAdes: a new versatile metagenomic assembler. *Genome Res*. 2017;27:824–34. https://doi.org/10.1101/gr.213959.116

76. Wu YW, Simmons BA, Singer SW. MaxBin 2.0: an automated binning algorithm to recover genomes from multiple metagenomic datasets. *Bioinformatics* 2016;32, 605–7. https://doi.org/10.1093/bioinformatics/btv638

77. Kang DD. et al. MetaBAT 2: an adaptive binning algorithm for robust and efficient genome reconstruction from metagenome assemblies. *PeerJ* 2019;7:e7359. https://doi.org/10.7717/peerj.7359

78. Chaumeil PA, Mussig AJ, Hugenholtz P, Parks DH. GTDB-Tk: a toolkit to classify genomes with the Genome Taxonomy Database. *Bioinformatics* 2019;36:1925–7. https://doi.org/10.1093/bioinformatics/btz848

79. Parks DH et al. A complete domain-to-species taxonomy for Bacteria and Archaea. *Nat. Biotechnol*. 2020;38:1079–86. https://doi.org/10.1038/s41587-020-0501-8

80. Davis NM, et al. Simple statistical identification and removal of contaminant sequences in marker-gene and metagenomics data. *Microbiome* 2018;6:226. https://doi.org/10.1186/s40168-018-0605-2

81. Podell S, Gaasterland T. DarkHorse: a method for genome-wide prediction of horizontal gene transfer. *Genome Biol*. 2007;8:R16. https://doi.org/10.1186/gb-2007-8-2-r16

82. Podell S. et al. Assembly-driven community genomics of a hypersaline microbial ecosystem. *PLoS One* 2013;8:e61692. https://doi.org/10.1371/journal.pone.0061692

83. Wickham H. ggplot2 : Elegant Graphics for Data Analysis. *Biometrics* 2016;67:678–9. https://doi.org/10.1007/978-3-319-24277-4_3

84. Fox J, Weisberg S. An R companion to applied regression. Los Angeles: Sage publications, 2018.
